# Mapping and accuracy assessment of siltation of recharge dams using remote sensing technique

**DOI:** 10.1038/s41598-020-67137-9

**Published:** 2020-06-25

**Authors:** Sankaran Rajendran, Sobhi Nasir, Khalifa Al Jabri

**Affiliations:** 1 Environmental Science Center, Qatar University, P.O.Box:2713 Doha, Qatar; 2 Earth Science Research Center, Sultan Qaboos University, Al-Khod, 123 Muscat, Oman; 3 Departments of Civil and Architectural Engineering, Sultan Qaboos University, Al-Khod, 123 Muscat, Oman

**Keywords:** Environmental impact, Environmental impact, Natural hazards, Natural hazards

## Abstract

This study demonstrates the use of ASTER data to map the siltation of the Al-Khod Dam constructed at the lower reaches of Samail Catchment area, north Muscat, Sultanate of Oman. In this study, the decorrelated image of spectral bands 6, 3, and 1 clearly discriminated the distribution of siltation. The occurrence of siltation is confirmed by the detection of minerals using the VNIR-SWIR bands by the Spectral Angle Mapper (SAM) method. In addition, the siltation of the dam is studied for the years 1987 to 2018 using Landsat satellite images. Interpretation of images showed a gradual increase of the siltation. Mapping accuracy for the occurrence of siltation is assessed by a confusion matrix which produced an overall accuracy of 97.88% and Kappa coefficient of 0.98 in the Maximum Likelihood method. Results of image analyses are verified in the field and confirmed through laboratory analyses. The study of spectral absorption characters of field samples using a Portable Infrared Mineral Analyzer (PIMA) showed the presence of OH molecule bearing minerals (clays, serpentines, etc.) and carbonate minerals (calcite and dolomite) in the silt deposits. The occurrence of these minerals is confirmed further by X-ray diffraction (XRD) and Scanning Electron Microscope (SEM) analyses.

## Introduction

Groundwater recharge in dams to increase the yield of groundwater for sustainability is very important in arid regions^1^. Heavy rainfall and occurrence of flash floods in the regions may deposit large amounts of silts in the dams^2–7^. The siltation is a major issue, hindering the infiltration of surface water into groundwater. Increase of such deposits reduce or stop groundwater recharge, decline groundwater levels, and well yields, and deteriorate water quality in and around the dam yearly^5,8^. Researchers described the groundwater mounds beneath recharge sites^9–11^ and infiltration during erratic floods in karstic basins^12–14^. An in-depth study of alluvial stratigraphy provides detail about the number of timing, events, and deposited volumes of floods^15,16^. A large number of check dams in the Mediterranean ephemeral streams have been built to prevent sediment inputs^17^. Sedimentation rates are studied by infill stratigraphy of check dam and a model is proposed to evaluate erosion and sediment yield processes^18,19^. However, meager attempts were made to study the siltations of the dams in Oman. Here the sedimentation in dams is influenced by numerous factors and the dams must be regularly cleaned and dried to continue the rates infiltration and storage capacities^4^. In the Sultanate of Oman, many recharge dams across wadis were constructed by the government to hold up runoff and recharge the aquifers. But, most of the dams are silted now due to wadi flow after flash floods in catchments. A suitable technique to map and assess the silt deposits and sedimentation, an alternative to expensive and time-consuming methods^20,21^, is essential and urgently needed by scientists and engineers to remove or protect the deposits of the recharge dams.

In this context, satellite images obtained using the remote sensing technique is helpful in the mapping of earth’s resources and monitoring of earth’s environments. The spectral bands of multispectral satellite sensors are capable of showing and mapping vegetation, minerals, rock formations, soils, and man-made constructions^22–25^. The technique is more suitable for mapping and study of siltation deposits of recharge dams found in the arid region since the region is directly exposed to the sensor of the satellite without any vegetation disturbance. Therefore, the objective of this study is to demonstrate the use of satellite data to map the siltation occurrence of a recharge dam found in the arid region. This study maps the siltation of the Al-Khod dam located at the southern Al Batinah coastal plain, north of Muscat, Oman using ASTER data, and assesses the distribution of siltations of the dam using Landsat data from 1990 to 2018.

### Recharge dams

**Figure 1:**
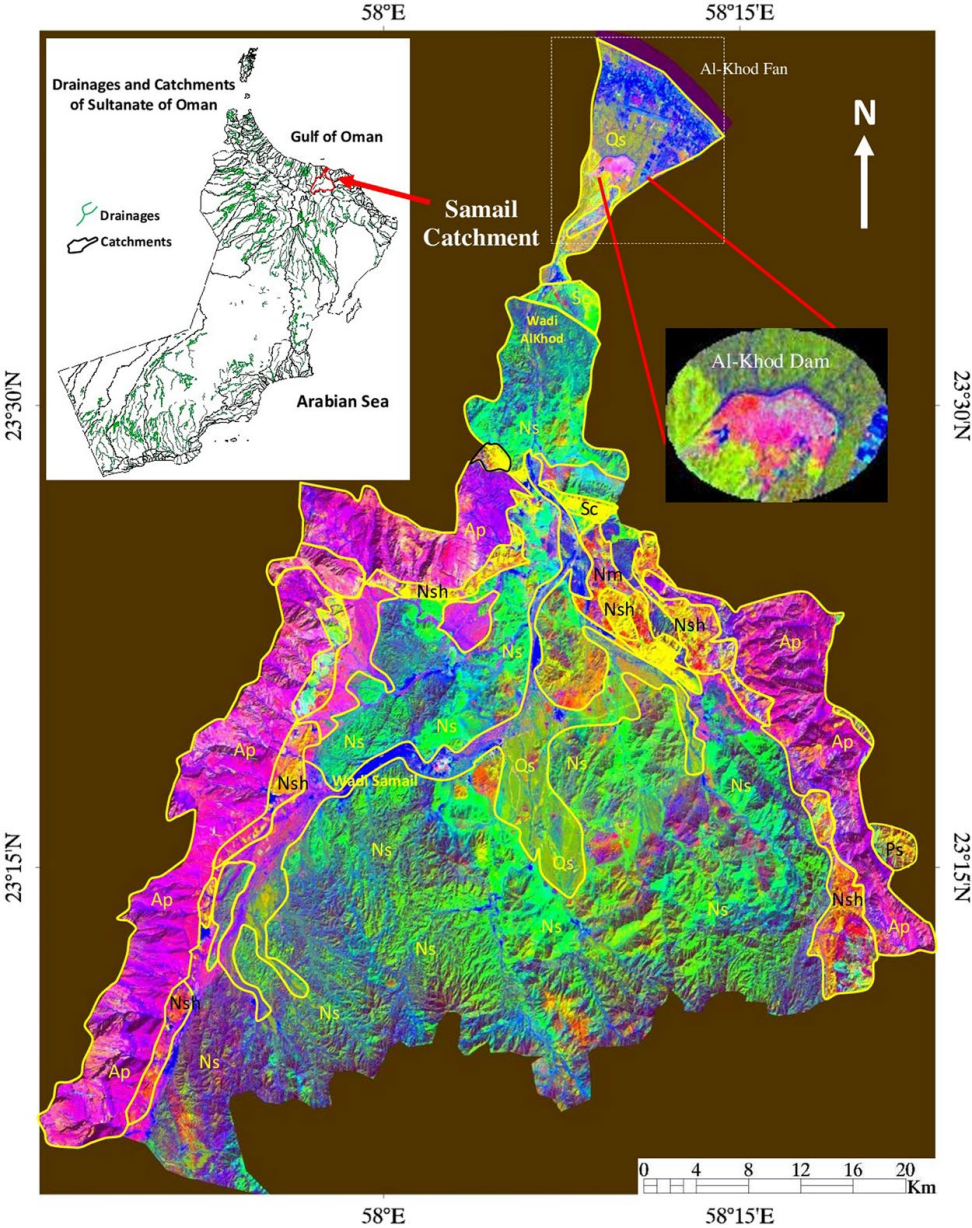
Samail catchment and location of the Al-Khod Dam situated across the Wadi Al-Khod in the decorrelated image of Landsat TM spectral bands 7, 5 and 4^21^ [ENVI 5.5 https://www.harrisgeospatial.com; https:// LPDAAC.usgs.gov]. Regional geology is incorporated^4^ [Qs: Quaternary surficial deposits; NS: Nappes – Samail ophiolite; Nsh: Nappes –Sumeini and Hawasina; Nm: Nappes – Metamorphic Sole; Ap; Middle Permian – Late Cretaceous Arabian Platform; Sc; End Cretaceous-Tertiaryry sedimentary cover; Ps: End Proterozoic – Early Permian Sedimentary basement].

In the Sultanate of Oman, the Ministry of Regional Municipalities and Water Resources (MRMWR) has constructed 34 recharge dams to increase groundwater potential^26^. 21 dams were constructed across the major wadis parallel to the Al Batinah coast of Oman for domestic and agricultural purposes. These dams vary in height (5–25 m), length (100–9,000 m) and storage capacity (0.04–11.4 Mm^3^)^26^. The Al-Khod Dam was constructed between December 1983 and March 1985 across Wadi Al-Khod in the lower reaches of the Samail Catchment at 23°38′ north latitudes and 58°10′ east longitudes (Fig. 1). The dam has a length of 5100 m, a maximum height of 11 m and a storage capacity of 11.6 Mm^3^. The estimated recharge of the dam is 3.38 Mm^3^/year^27^. It is an earth dam constructed by rock-fill gabions. The reservoir area of the dam is 3.2 km^2^. The major objectives of the construction of the dam are to increase aquifer’s recharge, provide an adequate water supply to adjacent regions, and to form a hydraulic barrier to mitigate seawater intrusion^5^. The Samail Catchment area of the dam covers an area of 1,635 km^2^ and flow by two main tributaries namely Wadi Samail and Wadi Al-Khod^6^. These ‘wadis’ drain water from the interior highlands towards the Al-Khod Fan (Fig. 1). The Al-Khod dam stores the water precipitated within the catchment, protect surrounding villages from small to medium floods^28^.

The area in and around of Al-Khoud dam receives annual rainfall more than 300 mm in the highlands to less than 100 mm in the coastal plain between June and September^5,29^. The hydrographs record of wadi gauging station located at 23°34′N latitude and 58°7′E longitude at an altitude of 78 m and a distance of 4 km upstream of the dam showed the occurrence of relatively two strong floods in 1996 and 1997^6^. The embankment of the dam was overtopped during three major floods that occurred during March 2007, June 2007 (Cyclone Gonu) and June 2010 (Cyclone Phet)^30,31^. The last two floods produced enormous damage to the populated area situated downstream of the dam. During which, the maximum flow rate of 178 m^3^/s was recorded at the gauging station and unfortunately, there are no data on the suspended loads, bed loads, or even the turbidity of water at the gauging station^6,31^. However, the periodic distribution of precipitation and the ephemeral nature of floodwaters deposit silts in the recharge dam and the arid climate keeps the dam dry in most of the months^4–6^, which permits researchers to carry out a study over the catchment and recharge dam^4–6^ and allows studying and monitoring the siltation of the dam using satellite data since the area is directly exposed to the sensor of the satellite without vegetation disturbance^25^.

**Figure 2:**
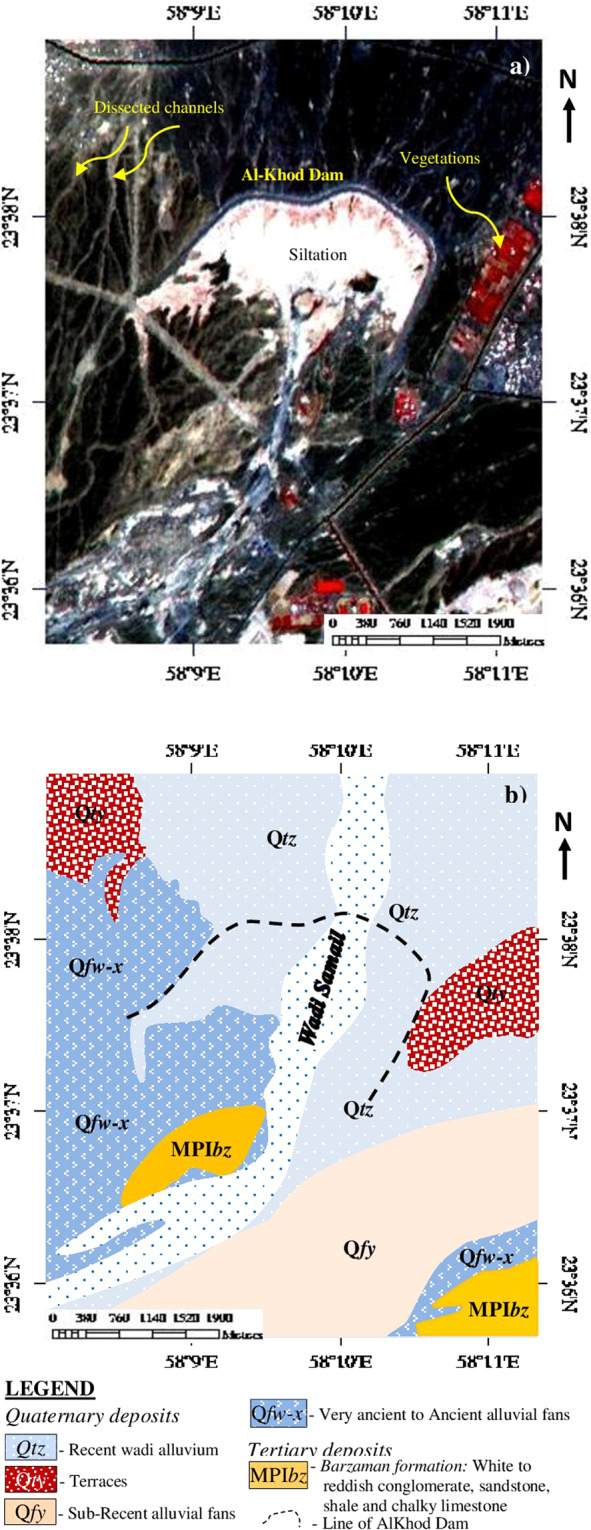
(**a**) ASTER image [R:3; G:2; B:1; ENVI 5.5 https://www.harrisgeospatial.com; https:// LPDAAC.usgs.gov] of the study area showing the Al-Khod Dam, siltation, dissected channels, and vegetation. (**b**) The Geology around the Dam.

Figure 2a, the ASTER RGB image (R:3, G:2, B:1) shows the siltation of the Al-Khod dam in white and the bund of the dam exhibits in dark. The distribution of vegetation in and around the dam appears in red. The geomorphology around the dam is plain and moderately dissected by channels and intermittent streams. The soils of the reservoir are alluvial and consist of limestone and ophiolite rocks derived from the mountains and upstream land^6,32^ (Fig. 1). Prior to the construction of the dam, the dominant soils of the AlKhoud dam area were carbonates, gypsum, sand, and silt^33^. The geology and hydrogeology of the underlying parent material of the dam are reported in detail^34,35^.

### Geology of the study area

Regional geology of Samail catchment shows the occurrence of major formations such as the sedimentary cover of End Cretaceous-Tertiary age (Sc), Arabian Carbonate Platform of Middle Permian – Late Cretaceous age (Ap), Samail ophiolite (NS) and surficial deposits of Quaternary age (Qs). The ophiolite sequence consists of dunite, harzburgite, gabbros, sheeted dykes and pillow lavas^5^. The geology overlaid on the decorrelated image of Landsat TM spectral bands (7, 5, 4) exhibits occurrence of the sedimentary cover in reddish yellow, Arabian Carbonate Platform in pink, ophiolites in pale green and the surficial deposits in yellowish green^36,37^ (Fig. 1). The recent wadi deposits are in blue. The detailed geology in and around the Al-Khod dam is given in Fig. 2b. The area consists of conglomerate, sandstone, shale and chalky limestone of Tertiary age (MPI*bz*), and the ancient and sub-Recent alluvial fans (Q*fw-x*, Q*fy*), terraces (Q*ty*) and Recent wadi alluvium of Quaternary age (Q*tz*)^38^.

### Satellite data

This study utilizes data of the Landsat series and ASTER. The data of ASTER sensor of Terra satellite is used for mapping of the siltation, rock types and minerals, since the ASTER has six bands in SWIR region characteristics to hydroxyl and carbonate spectral absorptions that allow mapping the silts, rocks, and minerals occurred in and around of the Al-Khod Dam. The study also uses the data of TM, ETM+ and OLI sensors of Landsat satellites to study the siltation of the dam for the years 1987 to 2018 since these sensors have continuity of data at 30 m spatial resolution for long periods.

In this study, ASTER Level 1B spectral bands of 29 March 2004, were acquired from NASA Land Processes Distributed Active Archive Center User Services, USGS Earth Resources Observation and Science (EROS) Center. Among the 14 bands, the nine VNIR–SWIR spectral bands (resampled to 15 m) were chosen as the area of interest and processed to map the siltation of the Al-Khod dam. The imageries have a cloud cover of 0%. The data were supplied with radiometric and geometric corrections applied. These are delivered in WGS84 Universal Transverse Mercator (UTM) projection (Zone 40 N, Clark 1980 spheroid, PSD93 datum, EPSG: 3440). During the pre-processing of the data sets, the atmospheric correction was applied to remove the spectral effects such as water vapor and aerosols. The raw radiance data from the imaging spectrometer was rescaled to reflectance data using Fast line-of-sight Atmospheric Analysis of Spectral Hypercubes (FLAASH) algorithm. A cross-track illumination correction using the ENVI 5.5 software (Harris Geospatial Solutions, Broomfield, CO, USA; https://www.harrisgeospatial.com) was carried out before the FLAASH data conversion. The siltation of Al-Khod dam of the years 1987, 1990, 2000, 2005, 2010, 2015 and 2018 was also studied using archived images of the TM, ETM+ and OLI sensors of Landsat satellites. The time-series data were pre-processed systematically to compare the acquired images. Initially, all images were resampled to a 30-m resolution. The images were coregistered using topographic maps (used to identify ground control points) and WGS84 UTM projection (Zone 40 N, Clarke 1980 spheroid, PSD93 datum, datum, EPSG: 3440) coordinate system with a root means square error of less than 0.5 pixels per image. Further, the images were processed systematically by converting DN values (recorded by the sensor) to top of atmosphere (TOA) reflectance and removal of atmospheric effects that occurred due to absorption and scattering (atmospheric correction). The DN values of images were converted to surface reflectance by the method consisting of sensor calibration^39–41^. A full radiative transfer modeling was applied including the correction of topography-induced illumination variations^39–41^. The impact of sensor degradation on the gain parameter and calibration were accounted using the data as described to ETM+ and TM sensors (tandem data sets)^42^, and OLI and ETM+ sensors (North African desert site)^43,44^. A radiative transfer model was performed for each scene based on the Acronym 6 S and 5 S Codes as described^45,46^. The atmospheric transmission factors were calculated using the Modtran-4 code^47^. All pre-processed data were studied to the area of interest using ENVI 5.5 image processing software. The sensors characters of ASTER, Landsat TM and Landsat ETM are given in Table 1. More details of the Landsat and ASTER satellites can be referred in http://landsat.usgs.gov/ and http://asterweb.jpl.nasa.gov/ respectively.

**Table 1:** The sensor characters of Landsat TM, ETM and ASTER instruments.

Sensors	Landsat5 TM	7ETM^+^	Landsat 8	Aster		
				VNIR	SWIR	TIR
Spectral	Band1 0.45–0.52	Band1 0.45–0.52	Band1 0.43–0.45 Coastal	Band 01 0.52–0.60	Band 04 1.6–1.7	Band 10 8.125–8.475
Resolution			Band2 0.45–0.51	Nadir looking		
with range (µm)	Band2 0.52–0.60	Band2 0.53–0.61	Band3 0.53–0.59	Band 02 0.63–0.69	Band 05 2.145–2.185	Band 11 8.475–8.825
			Band4 0.63–0.67	Nadir looking		
	Band3 0.63–0.69	Band3 0.63–0.69	Band5 0.85–0.88	Band 03N 0.76–0.86	Band 06 2.185–2.225	Band 12 8.925–9.275
			Band6 1.57–1.65	Nadir looking		
	Band4 0.76–0.90	Band4 0.75–0.90	Band7 2.11–2.29	Band 03B 0.76–0.86	Band 07 2.235–2.285	Band 13 10.25–10.95
			Band9 1.36–1.38 Cirrus	Backward looking		
	Band5 1.55–1.75	Band5 1.55–1.75	Band10 10.6–11.19	Band 08 2.295–2.365	Band 14 10.95–11.65	
		Band7 2.09–2.35	(TIRS1)			
	Band7 2.08–2.35	Band6 10.4–12.5	Band11 11.5–12.51	Band 09 2.36–2.43		
	Band6 10.40–12.50	(TIR)	(TIRS2)			
	(TIR)	PAN 0.52–0.90	Band8 0.50–0.68			
Spatial	30 m VNIR/SWIR	15 m PAN,	15 m PAN	15	30	60
Resolution (m)	120 m TIR	30 m VNIR/SWIR	30 m VNIR/SWIR			
	60 m TIR	100m TIR				
Temporal Resolution	16	16	16	16	16	16
Radiometric Resolution (bits)	8	9	16	8	8	12
Swath width (km)	180	185	185	60	60	60

*Landsat 7 ETM + : the Scan Line Corrector aboard malfunctioned on May 31, 2003. Data only in the middle part of the images can be used.

## Methods

The occurrence and distribution of siltation and the geological formations associated with the dam are carried out using ASTER bands 6, 3, and 1 and the decorrelation image classification method. This method is widely used and discussed^48–51^. Minerals of the silt deposits and associated formations are studied using nine VNIR-SWIR bands and spectral angle mapper (SAM) image classification method^52,53^. This method encompasses hyperspectral tools, such as Minimum Noise Fraction (MNF) transformation, Pixel Purity Index (PPI) and n-Dimensional visualizer, and classifies minerals of the image based on the collection of end-member spectral information^54–56^. The method used to the study area firstly determined the inherent dimensionality of MNF image data^57^ and showed increases of noise from MNF bands 1–9. These bands are further processed to determine most spectrally pure (extreme) pixels contain mineral information of the image by PPI. The PPI iteration value of 10,000 (the maximum), the threshold value of 2.5 and the SAM angle of 0.10 in radians were provided. The values set at the SAM procedure were classified the entire set of pixels of the image (124,310 pixels, total area of 27.97 Km^2^). The method produces a classified image based on the value you specify for SAM Maximum Angle. Decreasing this threshold usually results in fewer matching pixels (better matches to the reference spectrum). Increasing this threshold may result in a more spatially coherent image; however, the overall pixel matches will not be as good as for the lower threshold. The threshold value expresses the maximum acceptable angle for the separation between the end-member spectrum vector and the pixel vector in the number of bands of dimensional space^58^.

Further, the occurrence and spatial distribution of siltations were mapped for the years 1987, 1990, 2000, 2005, 2010, 2015 and 2018 by developing false-color composite (FCC) images (R:4, G:3, B:2) of the Landsat satellites^36,59^. The siltations are studied for spectral absorption characters and mapped using parallelepiped method^60,61^. In addition, an accuracy assessment for the distribution of siltation and associated formations of the study area is carried out by confusion matrix using the Maximum Likelihood (ML), and newest algorithms namely Spectral Angle Mapper (SAM) and Spectral Information Divergence (SID) to understand the ability among the methods^62–67^. All the processed images are interpreted and discussed and the results are verified in the field study and confirmed through laboratory analyses. The geological map of the area was used to interpret the results of image analyses and verify the geological formations in the field^38^. The samples collected during the fieldwork were studied for spectral characters of the silt deposits using a Portable Infrared Mineral Analyzer (PIMA) infrared spectrometer at the Earth Science Research Center, Sultan Qaboos University. The instrument is fabricated for field spectroscopy by Integrated Spectronics Pty Ltd., Australia. The spectral resolution of the instrument is ~7 nm. The instrument has a built-in wavelength calibration target plate to calibrate reflectance spectra and is capable to measure spectra from 10 s to around 5 min. speed. The instrument is provided with PIMA View Graph™ software (version 3.1; Integrated Spectronics Pty Ltd., Baulkham Hills, Australia) to process and study the spectra of the samples. The samples are also analyzed by XRD and SEM at the Central Analytical and Applied Research Unit (CAARU), Sultan Qaboos University and studied the minerals of the siltation.

## Results

### Mapping of siltation

**Figure 3:**
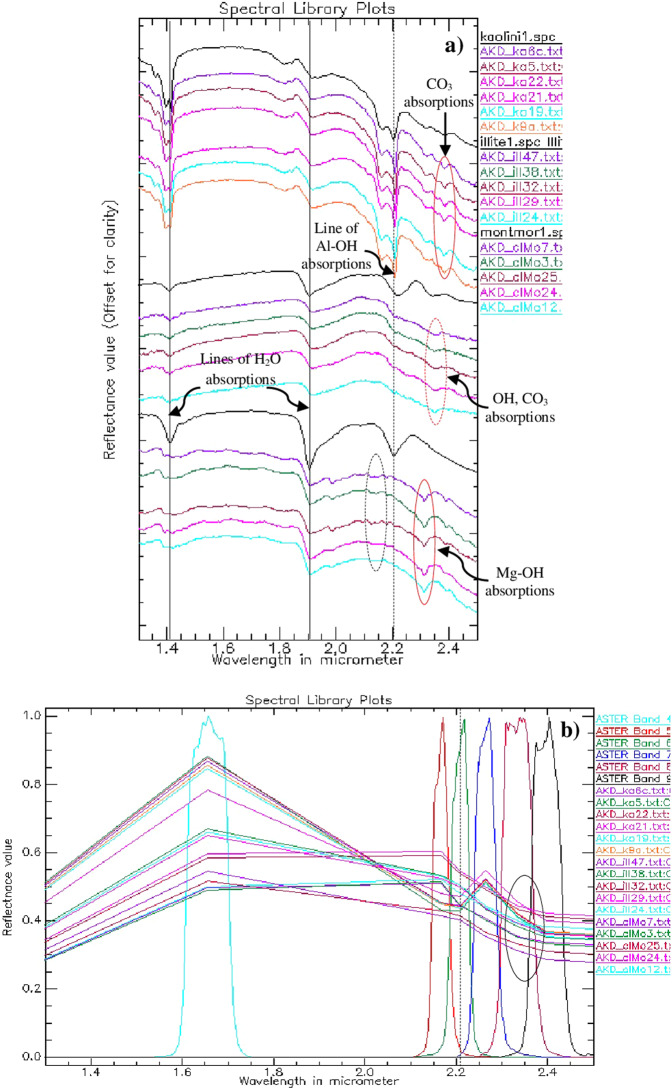
(**a**) Reflectance spectra of samples of the Al-Khod measured in 1.3 to 2.5 µm show H_2_O and hydroxyl molecules absorptions in 1.4 µm and 1.9 µm (black solid vertical lines), Al-OH absorption in 2.2 µm (black dashed vertical line and elliptical), Mg-OH absorption near 2.3 µm (red solid elliptical) and CO_3_ absorption near 3.35 µm (dashed elliptical). **(b)** Spectra of the samples resampled to the SWIR spectral bands of ASTER in 1.3 to 2.5 µm show Al-OH absorption in ASTER band 6 (black dashed vertical line) and Mg-OH and CO_3_ absorptions near ASTER band 8 (black solid elliptical).

In this study, the band 6 characteristics to Al-OH absorption is selected to map the siltation of the dam since the surface deposits consist of clays and silts^68–73^. Band 3 is chosen as albedo to characterize and highlight certain silicate minerals associated with the deposits and band 1 is preferred to discriminate iron-rich mineral-bearing weathered sedimentary sand and gravels, mainly the weathered harzburgite and gabbros transported from the catchment. The Al-OH absorption of spectral band 6 is studied by measuring spectra over field samples having kaolin (Al_2_Si_2_O_5_(OH)_4_) (AKD_K9a to AKD_Ka6c), illite ((K, H_3_O)(Al, Mg, Fe)_2_(Si, Al)_4_O10[(OH)_2_,(H_2_O)) (AKD_ill24 to AKD_ill47) and montmorillonite ((Na, Ca)_0,3_(Al, Mg)_2_Si_4_O_10_(OH)_2_·n(H_2_O)) (AKD_clMo12 to AKD_clMo7) using a PIMA spectrometer in the 1.3 to 2.5 µm wavelength (spectra stacked, Fig. 3a) and confirmed by resampling the spectra with the SWIR spectral bands of ASTER (Fig. 3b). The study of spectra showed two strong absorptions at 1.4 µm and 1.9 µm (black solid vertical lines) due to the presence of water and hydroxyl molecules^68,69^, absorption at 2.2 µm due to the presence of Al-OH^69,70^ (black dashed vertical line and elliptical), absorption at 2.3 µm due to the presence of Mg-OH^70–73^ (red solid elliptical) and absorption near 2.33 µm due to the presence of CO_3_^74–77^ (dashed elliptical) in the minerals of the samples (Fig. 3a). The spectra of the samples resampled to SWIR spectral bands of ASTER in the 1.3 to 2.5 µm showed the Al-OH absorption (black dashed vertical line) in ASTER band 6 and Mg-OH and CO_3_ absorptions (black solid elliptical) near ASTER band 8 (Fig. 3b).

**Figure 4:**
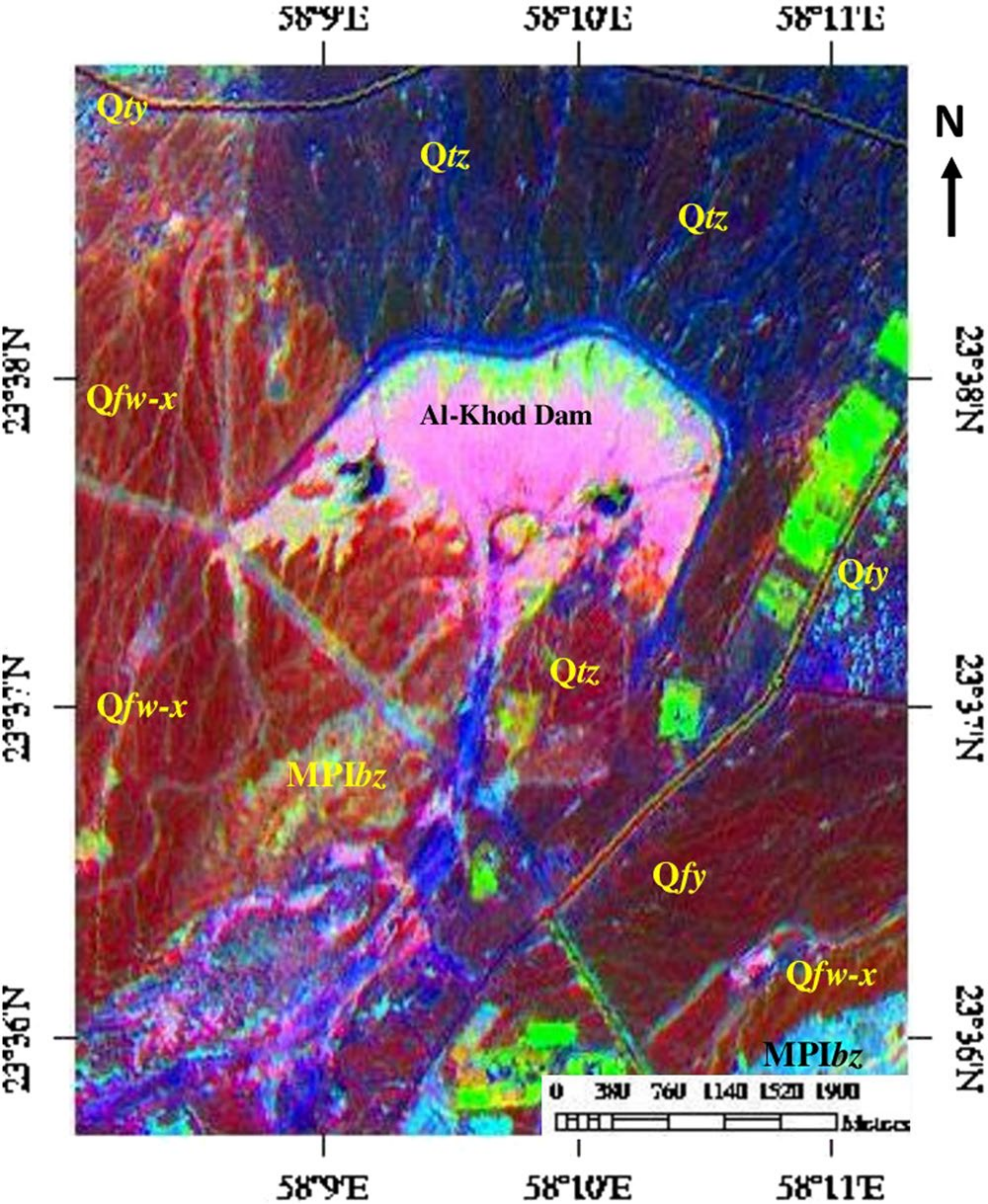
Decorrelated image of ASTER spectral bands 6, 3 and 1 [ENVI 5.5 https://www.harrisgeospatial.com; https:// LPDAAC.usgs.gov] shows the occurrence and distribution of siltation and different lithology in and around the Al-Khod Dam. [MPI*bz* – White to reddish conglomerate, sandstone, shale, and chalky limestone; Q*fw-x* – Very ancient to Ancient alluvial fans; Q*fy* – Sub-Recent alluvial fans; Q*ty* – Terraces; *Qtz* – Recent wadi alluvium].

The decorrelated image (R:6, G:3, B:1) is given in Fig. 4. The image clearly discriminated the siltation of the dam in pink. The dam has layers rich in clay and silt on the top, which exhibited in pink on the satellite data. The classification is due to the selection of spectral band 6, which is characteristic of absorption of water molecules and hydroxyl groups in the clay and silt minerals present in the dam deposits^6,68–71^. The sedimentary formations associated with the siltation and dam are well differentiated in different tones. The Tertiary deposit consist of conglomerate, sandstone, shale and chalky limestone (MPI*bz*) appear in cyan mixed with yellowish brown. The Ancient (Q*fw-x*) and sub-Recent (Q*fy*) alluvial fans and terraces (Q*ty*) of Quaternary deposits are exhibit shades of red to dark brown. The variations in tone are due to the spectral absorption of iron minerals in the weathered surface of sand and gravels. The Recent wadi alluvium (*Qtz*) appears in blue (Figs. 2b and 4). The vegetation of the area exhibits light green due to spectral absorption of water and chlorophyll contents in the vegetation.

### Mapping of minerals

**Figure 5:**
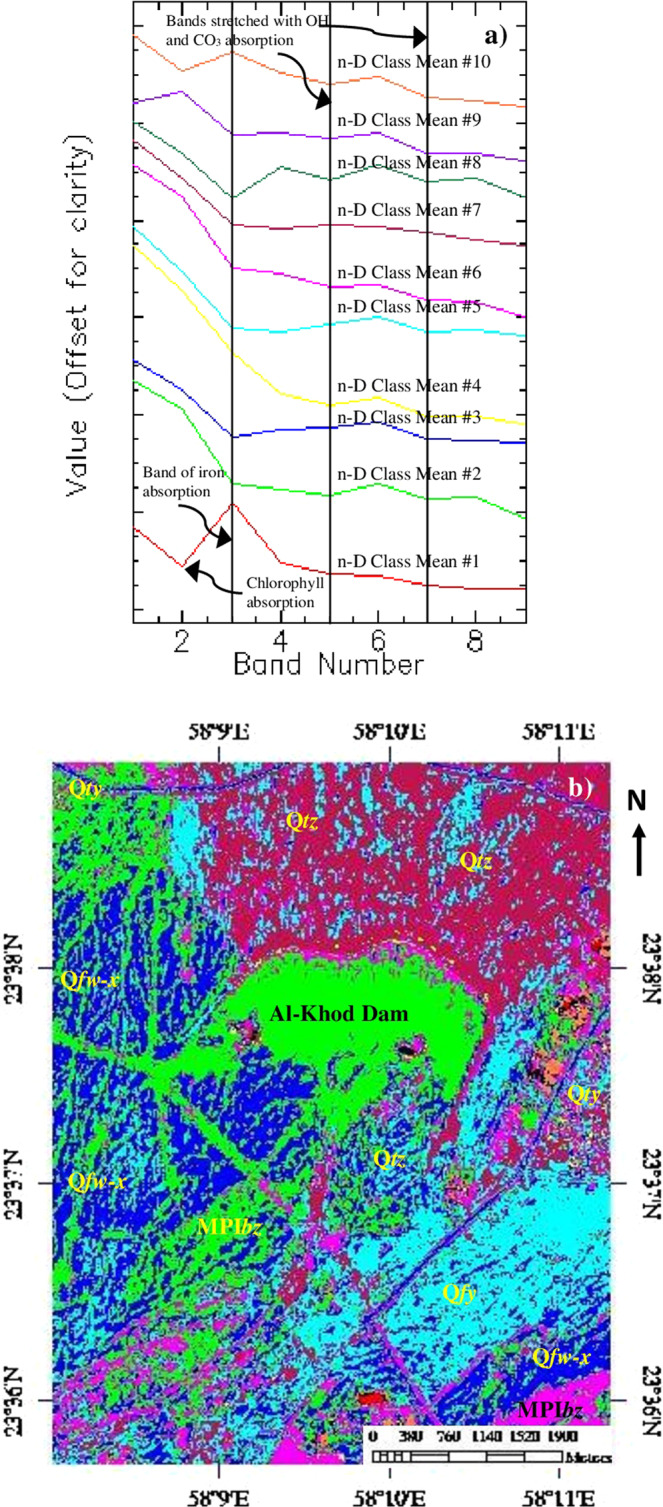
(**a**) Plot of endmember (stacked the Class Mean value against the VNIR-SWIR bands of ASTER) of SAM. (**b**) SAM image of the study area [MPI*bz* – White to reddish conglomerate, sandstone, shale and chalky limestone; Q*fw-x* – Very ancient to Ancient alluvial fans; Q*fy* – Sub-Recent alluvial fans; Q*ty* – Terraces; *Qtz* – Recent wadi alluvium; (ENVI 5.5 https://www.harrisgeospatial.com; https:// LPDAAC.usgs.gov)].

The results of the SAM classification are given in Fig. 5 and Table 2. Figure 5a shows the plot of endmembers (n-D class Mean). Table 2 provides details of the number of pixels of each class with their relative percentages. The total area of their distributions in the study area is provided in the table. Figure 5b is the SAM classified image that shows the occurrence and distribution of minerals of the siltation and the associated sedimentary formations. The classified image can be compared and better studied using Fig. 4, Table 2 and with the geological map (Fig. 2b).

**Table 2:** Distribution of pixels of study area in the SAM n-D classes.

n-D Classes^a^	No. of pixels	% in total area	Area in Km^2^
n-D Class Mean #1	554	0.446	0.12
n-D Class Mean #2	29520	23.746	6.64
n-D Class Mean #3	33458	26.915	7.53
n-D Class Mean #4	388	0.312	0.09
n-D Class Mean #5	19169	15.420	4.31
n-D Class Mean #6	14736	11.854	3.32
n-D Class Mean #7	25552	20.555	5.75
n-D Class Mean #8	321	0.258	0.07
n-D Class Mean #9	23	0.019	0.01
n-D Class Mean #10	589	0.474	0.13
Total	124, 310	100.000	27.97

a Unclassified: 0 points (0.000%) (0.0000 km^2^).

The image (Fig. 5b) shows the classified pixels mainly in five n-D classes viz. n-D class #2, #3, #5, #6 and #7 (Table 2; Fig. 5a,b). The pixels of n-D class #4, n-D class #8, n-D class #9 and n-D class #10 do not show significant mineral occurrences in the study area. The plot of n-D class #1 shows strong absorption in band 2 (0.63–0.69 µm) and flat absorptions from band 4 (1.6–1.7 µm) to band 9 (2.36–2.43 µm) (Fig. 5a) in the VNIR– SWIR regions. The pixels (0.12 km² - shown in red) are the classified vegetation and trees of the area found along the road (red in the FCC image, Fig. 2a and light green in the decorrelated image, Fig. 4). The absorption in band 2 is due to the absorption of chlorophyll contents in the vegetation and trees. The occurrences of such features in the area are verified during the fieldwork (See section Field studies).

The occurrence and distribution of carbonate minerals such as calcites and dolomites are detected in about 6.64 km^2^ (in green) by n-D class #2 and 7.53 km^2^ (in blue) by n-D class #3 respectively. The plot of n-D classes show absorptions mainly in bands 3 (0.76–0.86 µm), 5 (2.145–2.185 µm) and 7 (2.235–2.285 µm) (Fig. 5a) represent the influence of iron, MgOH, and CO_3_ contents^70–74^. The green pixels predominantly distributed in the whole Al-Khod dam and dissected channels due to the occurrence of clay and carbonate minerals. The detection of such pixels in the dam is due to overstretching of spectral information in the band 7. In addition, the green and blue pixels are found in the ancient alluvial fan region (Fig. 5b; Q*fy*) which is due to the presence of calcium and magnesium bearing minerals in the gravels of weathered harzburgites. The pixels of n-D class #5 (about 4.31 km^2^) in cyan are scattered in the Quaternary and Recent age alluvial deposits (Q*fy*, Q*ty*, and Q*tz*). The plot of class means show absorptions in the bands 3, 4 and 7. The absorption in bands 3 and 4 indicates the presence of iron and magnesium contents. The absorption in band 7 is due to the presence of OH contents in the altered aluminosilicate minerals such as amphibole, mica and chlorite groups present in the sands, gravels, and pebbles of the deposits^68–71,73^. The blue and green pixels are related to carbonate minerals.

The pixels of n-D class #6 (3.32 km^2^) and n-D class #7 (5.75 km2) appear in magenta and maroon respectively. These are related to the Tertiary formations (MPI*bz*), which consist of conglomerate, sandstone, shale, and chalky limestone, and the Recent wadi alluvium (Figs. 5b and 2b). The plot of n-D class #6 (Fig. 5a) shows absorptions in bands 3, 5 and 7. The absorptions in bands 3 and 5 are due to the presence of iron and magnesium bearing poorly altered minerals of olivine, pyroxene and amphibole groups^70–73^. The absorption in band 7 may be due to the scant occurrence of hydroxyl and carbonate contents in the minerals of the Tertiary formations (MPI*bz*)^71,76^. The plot of n-D class #7 show absorption in band 3, due to the presence of iron in the silicate minerals of olivine and pyroxene occurring in the unaltered harzburgite and gabbro deposited in the recent wadi alluvium (Q*tz*)^68–71,73^.

### Siltation from 1987 to 2018

**Figure 6:**
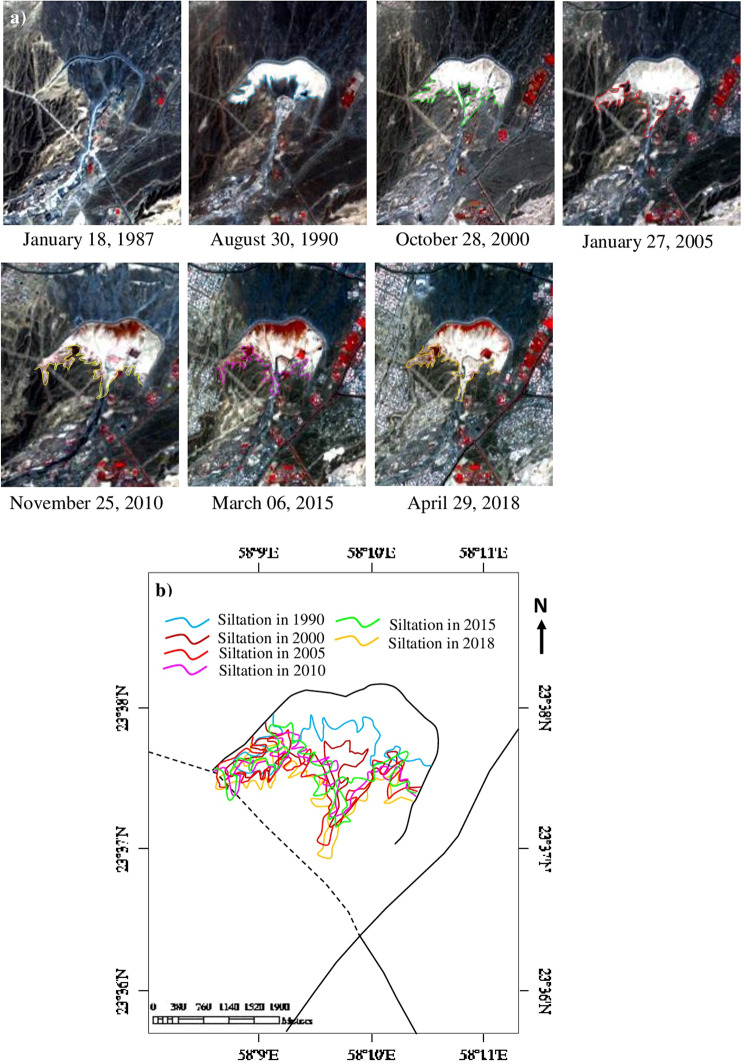
(**a**) RGB images [R:4; G:3; B:2; ENVI 5.5 https://www.harrisgeospatial.com] of Landsat satellites [https:// LPDAAC.usgs.gov] show the increase of siltation (white) and vegetation (red) from 1990 to 2018. (**b**) Year-wise increase of siltation in the Al-Khod Dam.

**Figure 7:**
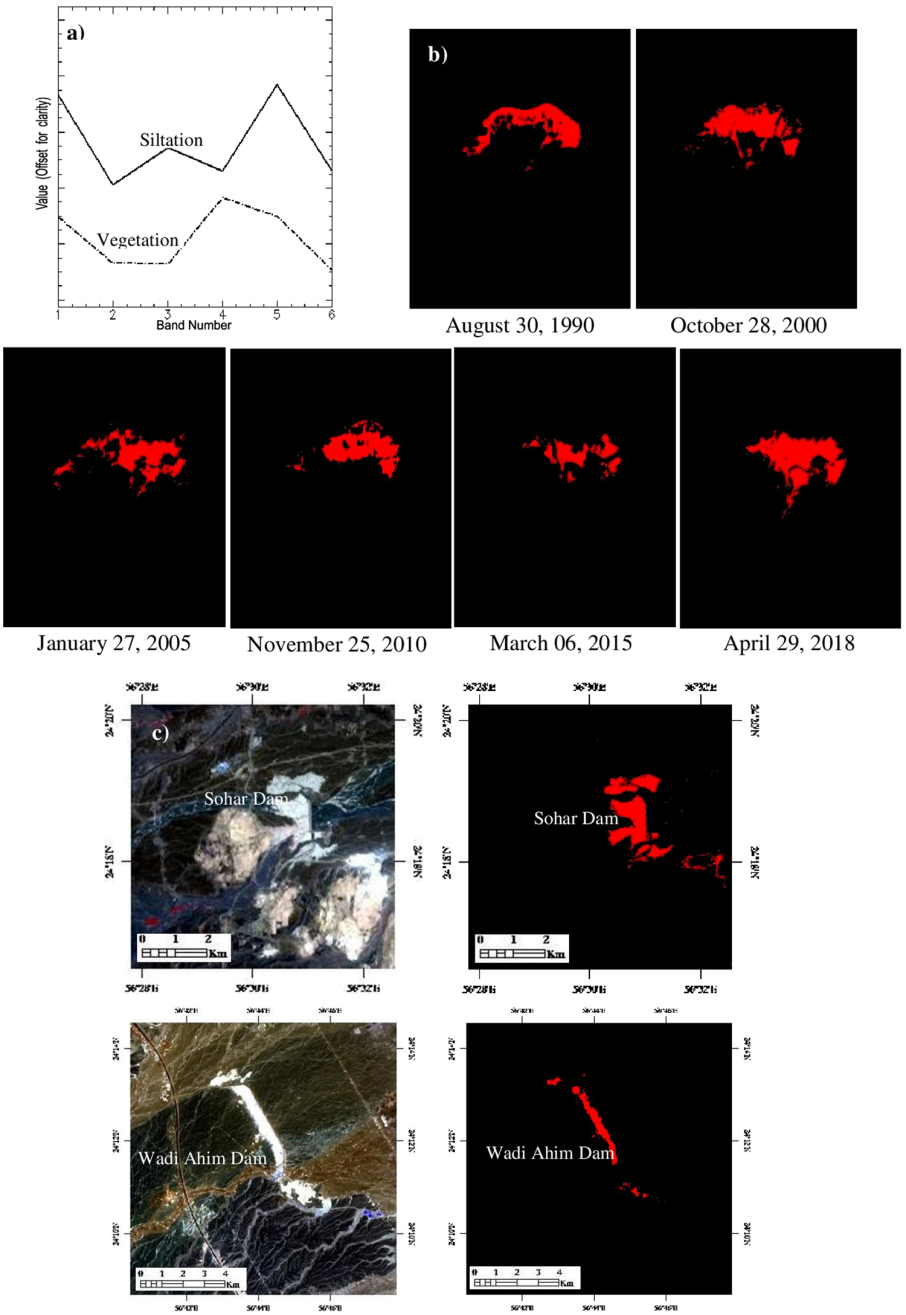
(**a**) Landsat 8 OLI image spectra [stacked by removing the aerosol, PAN, cirrus and thermal bands] of the siltation and vegetation. (**b**) Images classified by the Parallelepiped method show the occurrence and spatial distribution of siltation in the Al-Khod dam. (**c**) Occurrence and spatial distribution of siltation in the Sohar Dam and Wadi Ahim Dam [ENVI 5.5 https://www.harrisgeospatial.com; https:// LPDAAC.usgs.gov].

In this study, the siltation of Al-Khod dam of the years 1987, 1990, 2000, 2005, 2010, 2015 and 2018 are studied using archived images of the TM, ETM+ and 8 sensors of Landsat satellites (Fig. 6a; Table 3). The top layer rich in clay and silt in the dam exhibited a white tone on the satellite data and allowed to map the occurrence and spatial distribution of the siltation. The visual interpretation of false-color composites (Fig. 6a; R:4, G:3, B:2) of the year’s show changes occurred in the siltation. Figure 6b shows lines of the maximum distribution of the siltation of years. The images clearly show the occurrence and gradual increase in the distribution of siltation (white) and vegetation (red) in and around the dam from 1990 to 2018 (Fig. 6a,b; Table 3). The image of 1987 acquired after the construction of the dam (1985) shows no siltation or vegetation within the dam. The spectra collected over the Landsat 8 OLI image of the year 2018 (after removing the coastal aerosol, PAN, cirrus and thermal bands) for the siltation and vegetation is given in Fig. 7a as solid and dashed lines to understand the spectral absorption characters of the features. The spectra of the siltation show strong absorptions in bands 2, 4 and 6 (equivalent to ASTER spectral bands 1, 3, 5–8 respectively; Table 1). The absorptions in bands 2 and 4 are mainly due to the presence of weathered iron-rich silicate minerals in the boulders, gravels, and sands. The image spectra of the vegetation show strong absorptions in bands 2, 3 and 6. The absorptions in bands 2 and 3 are characteristic of the absorption of chlorophyll contents in the vegetation. The study of spectra clearly showed the absorptions of siltation and vegetation and confirmed their occurrences.

**Table 3:** The occurrence and spatial distribution of siltation and vegetation in Al-Khod Dam.

Sl. No.	Year	Landsat Sensors	Satellite data	Total area of siltation (Km^2^)	Occurrence and spatial distribution of	
					Siltation	Vegetation
1	18.01.1987	Landsat 5 TM	158r044_5dt19870118	No Siltation	No Siltation	No Vegetation
2	30.08.1990	Landsat 4 TM	L4158044_04419900830	1.47	Low Siltation	No Vegetation
3	28.10.2000	Landsat 5 TM	158r044_7dt20001028	2.18	Medium Siltation	Poor Vegetation
4	27.01.2005	Landsat 7 ETM +	L71158044_04420050127	2.55	High Siltation	Poor-medium Vegetation
5	25.11.2010	Landsat 7 ETM +	L71158044_04420101125	2.61	Very High Siltation	High Vegetation
6	03.06.2015	Landsat 8 OLI	LC81580442015063LGN00	1.61	Medium Siltation	Very High Vegetation
7	29.04.2018	Landsat 8 OLI	LC08_L1TP_158044_20180429_20180502_01_T1	2.83	Very High Siltation	High Vegetation

To assess the distribution of the siltation of the dam, the images of different years are mapped using the pixels (represented the spectra in Fig. 7a) by parallelepiped image processing method and the area of siltation is calculated (Table 2). The results of analyses for the years 1990, 2000, 2005, 2010, 2015 and 2018 are given in Fig. 7b, which show clearly the siltation of the Al-Khod Dam. The images exhibit the distribution of silt deposits in different directions and a gradual increase from the embankment of the dam. The image of 2015 shows the scarce distribution of siltation (Fig. 7b) due to the presence of the vegetation over silt deposits (Fig. 6b). The total area of siltation of each year in the dam is provided in Table 2, which show clearly the increase of siltation in the dam. In addition, the spectra were used in the parallelepiped method to map siltation of the Sohar Dam constructed across the Wadi Jizzi, near Sohar (latitude: 24°18′37.94″N; longitude: 56°30′53.04″E) and the Dam Ahin constructed across the Wadi Ahin between Saham and Sohar (latitude: 24°11′49.21″N; longitude: 56°44′26.18″E) using the Landsat 8 OLI data (LC08_L1TP_159043_20180506_20180506_01_RT). The results of the analysis are given in Fig. 7c. The spectra and the image processing method used to map the siltation of the dams clearly classified in Fig. 7c (in red in the classified image). This classification may depend on the resolutions of the sensors and the capability of the image processing method. This study shows the capability of the ASTER sensor to map siltation and detect minerals of the siltation that occurred in a recharge dam. As well as, the mapping of siltation over the Landsat images of the years 1990, 2000, 2005, 2010, 2015 and 2018 showed the increase in siltation in the Al-Khod Dam. The semi-qualitative results obtained from this study provided information useful to assess the siltation of the dam. However, a detailed large-scale field survey is recommended for remedial measures of the dam.

### Accuracy assessment

Researchers have used image processing algorithms over satellite data to assess the accuracy of geological mapping^62–67^. In this study, mapping of siltation is assessed by confusion matrix using Maximum Likelihood (ML), Spectral Information Divergence (SID) and Spectral Angle Mapper (SAM) image classification algorithms. The confusion matrix provides accuracy by comparing a classification result with ground truth information such as a ground truth image or ground truth ROIs. This procedure computes and provides results of overall accuracy, producer and user accuracies, Kappa Coefficient, and errors of commission and omission (ENVI 5.5). In this study, the ground truth based ROI’s are used for accuracy assessment since the library and field spectra were not obtained under the same conditions as the satellite images acquired^66,67,73^. The image endmembers are directly associated with surface components detectable in the image. However, the spectral absorption characters of the endmembers extracted from the image by PPI algorithms (Fig. 5a), the image spectra (Fig. 7a), the spectra of USGS Spectral Library for minerals^78^ and the spectra measured over field samples using a PIMA spectrometer (Fig. 3a,b) are studied while selecting the ROI’s. More details of the algorithms are also reported^52,63–67^.

Confusion matrices of the said algorithms are carried out to the subset (310 ×401 pixels of the data of 2018) of the study area. The results of the matrices are provided in Table 4. It shows the best accuracy of 97.88% % and the Kappa Coefficient of 0.98 produced by ML algorithm while comparing it to the SID and SAM algorithms. The SID and SAM algorithms provided accuracies of 95.76% and 84.85%, and Kappa Coefficients of 0.95 and 0.82 respectively (Table 4). The matrices of ML and SID algorithms showed clearly the occurrence of siltation with the highest User’s accuracy of 100%. The classification may be characteristic of the simple compositions (clay and carbonates) of the siltation. The other sedimentary formations produced User’s accuracies from 80% to a maximum of 100%. It is interesting to note that the Recent wadi alluvium (Q*tz*) and Sub-Recent alluvial fans (Q*fy*) produced a User’s accuracy of 100% compared to the Ancient or Tertiary deposits, which may be due to the absorption characters of such deposits. Both the algorithms have similar User’s and Producer’s accuracies and differ from the accuracies of the SAM algorithm. The difference in the SAM results may be due to the classification which is based on the laboratory spectra. All algorithms provided 100% User’s and Producer’s accuracies to map the vegetation. The algorithm classified the siltation of Al-Khod Dam clearly and differentiated from the associated sedimentary formations. No significant larger commission and omission errors were observed in the confusion matrix. On the other hand, the SAM identified the siltation and sedimentary formations with different User’s accuracies from 45% to 92.11%, with exception to Sub-Recent alluvial fans (Q*fy*) which produced 100% accuracy (Table 4).

**Table 4:** Accuracy assessment matrix of ML, SID and SAM algorithms applied to the training classes (Siltation – silt deposits; Qtz - Recent wadi alluvium; Qfw-x – Very ancient to Ancient alluvial fans; Qfy – Sub-Recent alluvial fans; Qty – Terraces; MPIbz – white to reddish conglomerate, sandstone, shale and chalky limestone; Vegetation – plantation and agriculture).

Class	Siltation	Qtz	Qfw-x	Qfy	Qty	MPIbz	Vegetation
Accuracy assessment matrix of ML algorithm.							
User’s accuracy	100.00	100.00	80.00	100.00	96.00	98.12	100.00
Producer’s accuracy	100.00	100.00	100.00	87.50	100.00	91.43	94.44
Accuracy assessment matrix of SID algorithm.							
User’s accuracy	100.00	100.00	80.00	100.00	94.00	94.12	100.00
Producer’s accuracy	100.00	100.00	100.00	87.50	100.00	91.43	94.44
Accuracy assessment matrix of SID algorithm.							
User’s accuracy	45.00	77.78	72.73	100.00	92.11	87.88	100.00
Producer’s accuracy	100.00	100.00	100.00	87.50	74.47	82.86	86.11

### Field studies

Although the images of ASTER and Landsat sensors used in this study were recorded in different years, the mapping of siltation and associated formations of the Al-Khod dam carried out was evaluated through several fieldworks conducted in the study area. During the field studies, traverse based samples were collected for laboratory analysis to confirm the silt mineralogy.

**Figure 8:**
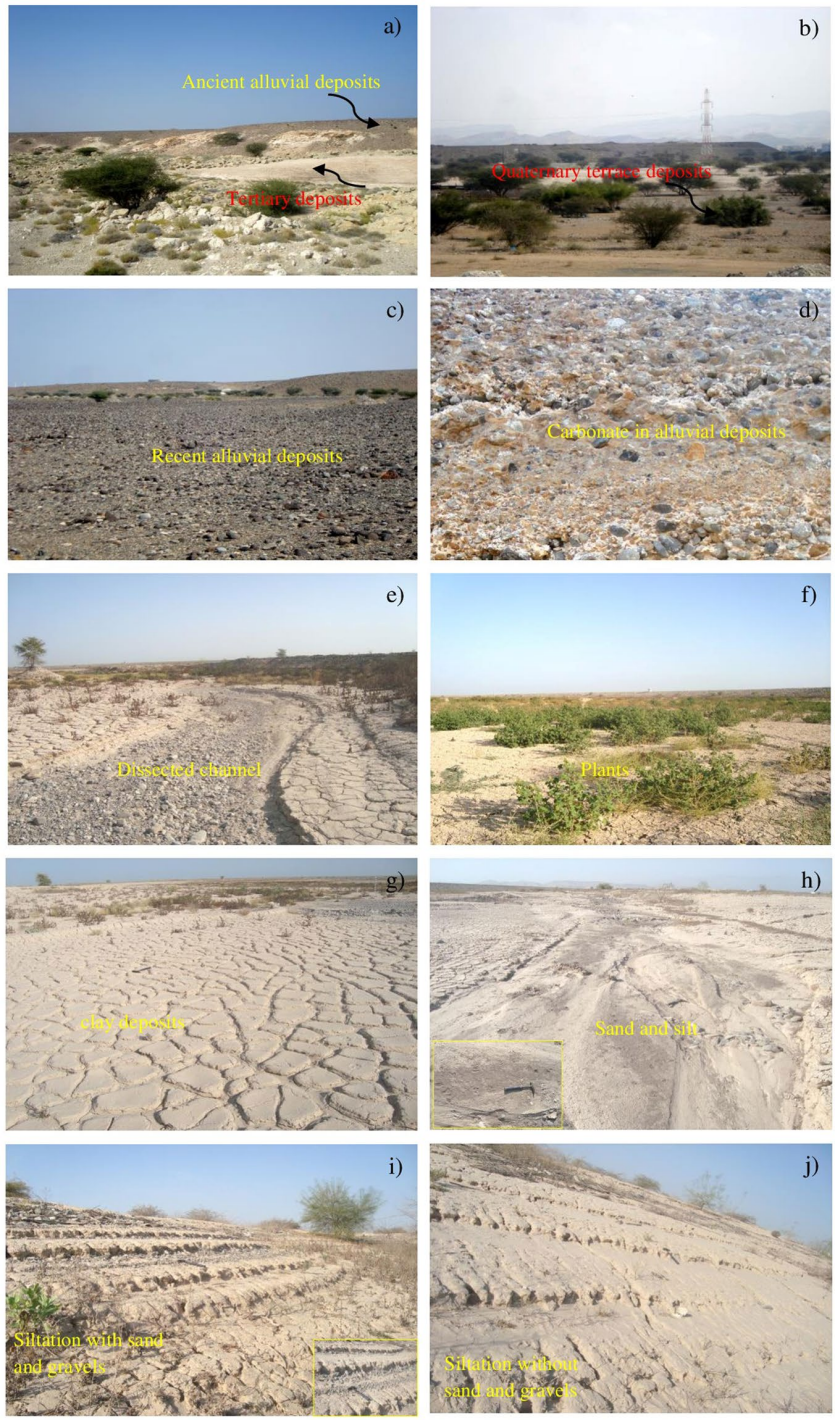
Field photographs showing the occurrence of (**a**) the Tertiary deposits (bottom) overlain the Ancient alluvial deposits (top), (**b**) the Terraces, (**c**) the Recent wadi alluvium, and the presence of (**d**) the carbonate in the alluvial deposits, (**e**) the dissected channels, (**f**) the scrubs and plants, (**g**) the clay deposits, (**h**) the sand with silt deposits, (**i**) the layers of the silt deposits with more than 0.5 m thickness consist of the sand and gravels (inset is graded bedding in siltation) and (**j**) the layers of the silt deposits with more than 0.5 m thickness without the sand and gravels occurred in and around of the Al-Khod dam.

In the study area, the occurrence of white to reddish conglomerate, sandstone, shale and chalky limestone (Fig. 8a; 23°36′47.99″N: 58°10′1.36″E) of Tertiary age, and of Ancient and Sub-Recent alluvial fans, terraces (Fig. 8b; 23°37′22.73″N: 58° 9′1.84″E), and recent wadi alluvium (Fig. 8c; 23°36′53.94″N: 58° 9′37.06″E) of Quaternary age were checked in order to verify the processed satellite images. The Quaternary deposit contains gravelly well-sorted sediments in the drainage system. A mixture of well-sorted boulders, gravels, and sands with minor clays and carbonates was noted. Carbonates were observed in the ancient alluvial deposits (Fig. 8d; 23°37′21.44″N: 58° 9′37.97″E). The presence of dissected channels with plant growths (Fig. 8e; 23°37′27.04″N: 58° 9′42.33″E) in the study area is verified.

The reservoir surface has a covering of vegetation (scrubs and plants, Fig. 8f; 23°37′41.94″N: 58°10′5.08″E), low-permeability cake or siltation consisting of clay and carbonates (Fig. 8g: 23°37′52.83″N: 58°10′16.93″E) and sand with silt deposits (Fig. 8h; 23°37′21.81″N: 58° 9′50.64″E). The siltation occurs as a sequence of layers, with graded bedding having a thickness of more than 3 m, as observed in certain areas of excavated vertical section (Fig. 8i,j; 23°37′34.43″N: 58°10′9.00″E). These represent the siltations of different periods^6^. The grain size of these deposits decreases towards the embankment of the dam where the clay and silt content increases. The sands, gravels, and boulders are cemented with fine carbonate and clay materials, causing a reduction of porosity, permeability, and infiltration rate. The necessary efforts taken by the Ministry of Regional Municipalities and Water Resources, which operate the dam, at the geotechnical remedy to remove the siltation are clearly witnessed in the field by scraped surfaces, local depressions, and excavations^6^. Scrub, bushes, and plants are observed in the dam where the sand is mixed with silt deposits (Fig. 8f).

### Laboratory analyses

Field **s**amples have been studied in the laboratory using a PIMA spectrometer for the understanding of spectral absorption characters of the silt deposits, and a Scanning Electron Microscope (Jeol JSM 7600 F) and X-ray diffraction methods to confirm the minerals of the siltation.

**Figure 9:**
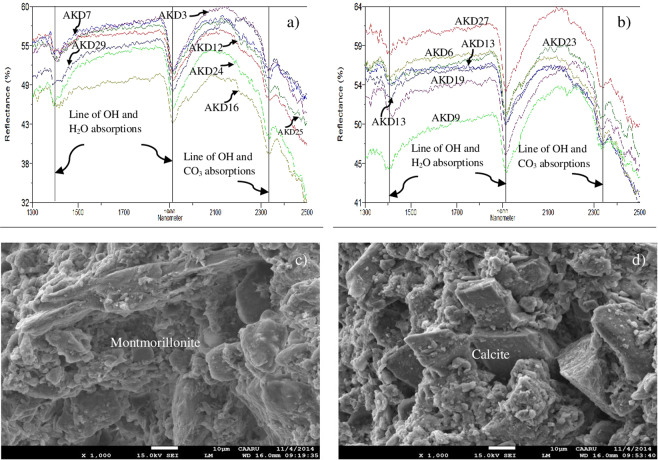
PIMA spectral plots showing the absorption regions of (**a**) the montmorillonite and antigorite, (**b**) the montmorillonite and calcite minerals; SEM images showing the presence of (**c**) the montmorrillonite (AKD16) and (**d**) the calcite (AKD27) minerals in the siltation of Al-Khod Dam.

**Figure 10:**
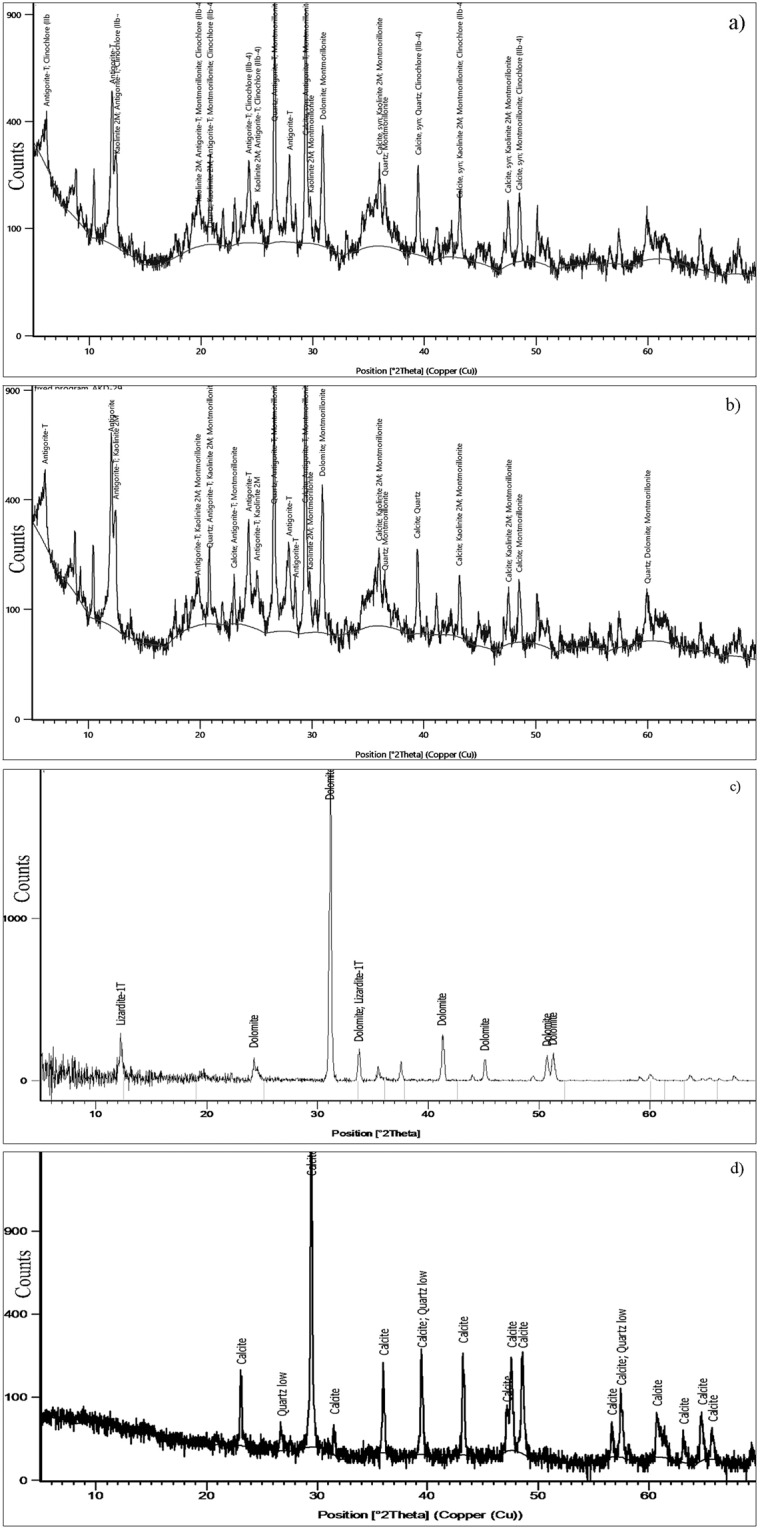
X-Ray diffraction analyses showing (**a–d**) the presence of OH bearing kaolinite, montmorillonite, antigorite, lizardite, and clinochlore minerals, and the carbonate-bearing calcite and dolomite minerals in the siltation of Al-Khod Dam.

More than two hundred and fifty spectral measurements were collected at different locations in the dam and over the samples collected in the field in the laboratory using the spectrometer. Selected spectra are given in Fig. 9a,b. The plots correlate well with the studied spectra (Figs. 5a and 7a). It shows absorptions near 1400 nm and 1900 nm due to the absorptions of water and hydroxyl molecules and at around 2300 nm due to the absorption of Mg-OH and CO_3_ molecules that occurred in the minerals of the siltation. The strong absorptions at around 1900 µm are due to the presence of hydroxyl minerals, namely montmorillonite ((Na, Ca)_0,3_(Al, Mg)_2_Si_4_O_10_(OH)_2_·n(H_2_O)), halloysite (Al_2_Si_2_O_5_(OH)_4_), illite ((K, H_3_O)(Al, Mg, Fe)_2_(Si, Al)_4_O10[(OH)_2_,(H_2_O)), kaolinite (Al_2_Si_2_O_5_(OH)_4_) and antigorite ((Mg, Fe^++^)_3_Si_2_O_5_(OH)_4_). The absorptions at around 2.3 µm are due to carbonate minerals, namely calcite (CaCO_3_) and dolomite (CaMgCO_3_) in the siltation. The hydroxyl minerals associated with carbonate minerals were detected in the samples. To confirm the presence of such minerals, the selected samples (AKD16 and AKD27) were coated with platinum and studied by the SEM analysis method. The study shows the presence of OH bearing Montmorillonite (Fig. 9c) and halloysite minerals and carbonate-bearing dolomite and calcite (Fig. 9d) minerals. Moreover, the presence of minerals is confirmed by using XRD analysis in the laboratory. The result of analyses shows the presence of montmorillonite, lizardite, antigorite, dolomite and calcite minerals (Fig. 10a–d). The sieve analyses of selected samples of the Al-Khod dam showed the presence of sands, silts, and clays in the ranges from 67.4 to 95, 25.1 to 5 and 7.5 to 4.5%p respectively (Table 5). The results confirmed the presence of clay and silt rich top layers with coarse sands at the bottom (Fig. 8e,i)^6^. The field study showed that the dam has coarse gravels and fragments below the siltation (Fig. 8i).

**Table 5:** Results of grain size analyses of samples of the Al-Khod Dam shows distribution of sands, silts and clays in the silt deposits.

Sample No	Sand %	Silt %	Clay %
K1	77	16.5	6.5
K2	81	14.5	4.5
K3	67.4	25.1	7.5
K4	95	5	0

## Discussion and Conclusions

In this study, the mapping of the siltation of Al-Khod Dam is carried out using ASTER data by the decorrelated stretching image classification method. The selection of spectral band 6 which is characteristic to absorption of OH molecules of the silt and clay minerals in the silt deposits well discriminated the siltation of the dam in pink in the decorrelated image (R:6, G:3, B:1; Fig. 4). The absorption of the spectral band is studied by measuring spectra over field samples having kaolin, illite, and montmorillonite using a spectrometer in the 1.3 to 2.5 µm wavelength and confirmed by resampling the spectra with the SWIR spectral bands of ASTER (Fig. 3). The study of spectra of samples showed two spectral absorptions at 1.4 µm and 1.9 µm due to the presence of water and hydroxyl molecules, an absorption at 2.2 µm due to the presence of Al-OH, absorption at 2.3 µm due to the presence of Mg-OH, and absorption near 2.33 µm due to the presence of CO_3_ in the minerals of the samples. The spectra of the samples resampled to SWIR spectral bands of ASTER in the 1.3 to 2.5 µm showed the Al-OH absorption in ASTER band 6 and Mg-OH and CO_3_ absorptions near ASTER band 8. The classification method well discriminated the sedimentary formations and vegetation that are associated with the siltation. The study of silt minerals in the dam by the SAM method showed the presence of OH and CO_3_ bearing clay and carbonate minerals (Fig. 5). The method also detected the minerals of iron and magnesium-rich aluminosilicate that are found in the highly weathered and poorly altered sands, gravels and pebbles of harzburgites and gabbros of the ophiolitic rocks based on their spectral absorption characters. The absorptions of minerals are studied in the spectral bands using the plot of n-D classes.

The visual interpretation of false-color composite images (R:4; G:3; B:2; Fig. 6a) of the years 1987, 1990, 2000, 2005, 2010, 2015 and 2018 showed an increase in the siltation. The image of 1987 acquired after the construction of the dam (1985) shows no siltation or vegetation within the dam. The study of the distribution of siltation by the parallelepiped image processing method confirmed the distribution of silt deposits and increases from the embankment of the dam (Fig. 7). The study of Landsat images showed clearly the changes in the occurrence and spatial distribution of siltation in the dam. The increase of siltation may depend on the amount of precipitation and silt carried from the catchment by wadi flows/floods from year to year. The siltation of the year 2015 is relatively low compared to the year 2000, 2005, 2010 and 2018, which is due to the occurrence of vegetation over the deposits. A similar study carried out to the Sohar Dam and Ahim Dam supported the image processing method to map the siltation of the dams. This study assessed the accuracy of siltation mapping by confusion matrix using Maximum Likelihood (ML), Spectral Information Divergence (SID) and Spectral Angle Mapper (SAM) image classification algorithms. The study of accuracy assessment of siltation showed that the ML and SID algorithms best distinguished the siltation of the Al-Khod dam (with the highest User’s accuracy of 100%) and the associated sedimentary formations (Table 4). The ML algorithm seems most successful in identifying the siltation and other formations.

The occurrence of conglomerate, sandstone, shale and chalky limestone of Tertiary age, and Ancient and Sub-Recent alluvial fans, terraces, and recent wadi alluvium of Quaternary age were checked (Fig. 8). The reservoir surface has low-permeability siltation consisting of clay, carbonates, sand and silt deposits. The siltation found as a sequence of layers with graded bedding which represents the siltations of different periods (Fig. 8f–j). The study of field samples using the PIMA spectrometer showed the spectral absorptions around of 1400 nm and 1900 nm due to the absorption of water and hydroxyl molecules, and at around 2300 nm due to the absorption of Mg-OH and CO_3_ molecules that occur in the minerals of the siltation (Fig. 9a,b). The analysis samples under SEM and XRD methods confirmed the presence of montmorillonite, halloysite, dolomite and calcite minerals in the siltation (Figs. 9c,d, 10a–d). The grain size analyses of samples of the dam indicated the presence of different sediment loads inside the dam and in the off-dam adjacent zones, both upstream and downstream of the embankment^6^. However, a detailed sub-surface study is recommended to obtain a quantified plan of the siltation affected areas, which may guide the removal of siltation to increase groundwater recharge.

Overall, the analyses of the ASTER and Landsat data showed their ability to map and monitor the occurrence of the siltation of the Al-Khod dam. The decorrelated stretching method is successful in discriminating the siltation and associated sedimentary formations. The SAM method is able to detect the minerals of the siltation and the other formations. The accuracy assessments study for the mapping of siltation by confusion matrix using different image classification algorithms showed that the ML and SID algorithms best distinguished the siltation of the Al-Khod dam and the associated sedimentary formations of the study area. The qualitative and semi-quantitative analyses of the ASTER and Landsat data of different years showed the occurrence and increase of siltation in the dam clearly. The accuracy analyses of the mapping methods supported the image processing methods to map the siltation of the dams. All the images of methods were studied and verified in the field. The spectral characters of the clay deposits were studied using a spectrometer and the presence of minerals of the siltation was confirmed by SEM and XRD analyses. This study demonstrated the sensor capability of ASTER and Landsat satellites and showed the potential of the well-known image processing methods to map the siltation of Al-Khod Dam and the sedimentary formations of the study area and to detect the minerals of the siltation and associated formations.
